# Anti-Biofilm Activity of a Low Weight Proteinaceous Molecule from the Marine Bacterium *Pseudoalteromonas* sp. IIIA004 against Marine Bacteria and Human Pathogen Biofilms

**DOI:** 10.3390/microorganisms8091295

**Published:** 2020-08-25

**Authors:** Ibtissem Doghri, Emilie Portier, Florie Desriac, Jean Michel Zhao, Alexis Bazire, Alain Dufour, Vincent Rochette, Sophie Sablé, Isabelle Lanneluc

**Affiliations:** 1 LIENSs UMR 7266 CNRS-Université La Rochelle, Université de La Rochelle, 17000 La Rochelle, France; ibtissem.doghri@umontreal.ca (I.D.); emilie.portier@univ-ubs.fr (E.P.); qiuyu.zhao@univ-lr.fr (J.M.Z.); vincent.rochette19@gmail.com (V.R.); sophie.sable@univ-lr.fr (S.S.); 2 LBCM EA3884, Institut Universitaire Européen de la Mer, Université de Bretagne-Sud, 56100 Lorient, France; floriedesriac@hotmail.fr (F.D.); alexis.bazire@univ-ubs.fr (A.B.); alain.dufour@univ-ubs.fr (A.D.)

**Keywords:** anti-biofilm, marine bacteria, *Pseudoalteromonas*, *Roseovarius*, P_004_ proteinaceous molecule, pathogenic bacteria

## Abstract

*Pseudoalteromonas* bacteria are known as potential bioactive metabolite producers. Because of the need to obtain natural molecules inhibiting the bacterial biofilms, we investigated the biofilm inhibitory activity of the marine bacterium *Pseudoalteromonas* sp. IIIA004 against the pioneer surface colonizer *Roseovarius* sp. VA014. The anti-biofilm activity from the culture supernatant of *Pseudoalteromonas* sp. IIIA004 (SN_IIIA004_) was characterized in microtiter plates (static conditions/polystyrene surface) and in flow cell chambers (dynamic conditions/glass surface). The *Pseudoalteromonas* exoproducts exhibited an inhibition of *Roseovarius* sp. VA014 biofilm formation as well as a strong biofilm dispersion, without affecting the bacterial growth. Microbial adhesion to solvent assays showed that SN_IIIA004_ did not change the broad hydrophilic and acid character of the *Roseovarius* strain surface. Bioassay-guided purification using solid-phase extraction and C_18_ reverse-phase-high-performance liquid chromatography (RP-HPLC) was performed from SN_IIIA004_ to isolate the proteinaceous active compound against the biofilm formation. This new anti-biofilm low weight molecule (< 3kDa), named P_004_, presented a wide spectrum of action on various bacterial biofilms, with 71% of sensitive strains including marine bacteria and human pathogens. *Pseudoalteromonas* sp. IIIA004 is a promising source of natural anti-biofilm compounds that combine several activities.

## 1. Introduction

In the marine environment, submerged surfaces are the subject of active bacterial colonization. Once attached to the substratum, the bacterial communities rapidly form biofilms and secrete extracellular polymeric substances (EPS) which are major components of the biofilm matrix. Rich in polysaccharides, proteins, lipids, DNA, RNA, and water, this matrix protects the microbial cells against stress, antibiotics, host immune system, and insures the stabilization of biofilms [1,2,3,4].

Biofilms are involved in several infectious diseases, both in humans and animals, and are present in a wide range of ecosystems, such as food industries, medical equipment, and natural environments [5,6,7]. In the marine environment, biofilms on submerged surfaces serve as reservoirs for pathogenic bacteria, from which they can disseminate [8]. Moreover, biofilms can damage maritime infrastructures through biocorrosion [9]. Fouling of ship hulls has also an important economic impact due to increased fuel consumption and maintenance costs [10]. The development of new strategies for the prevention and the treatment of adhesion and biofilm formation is therefore essential. The traditional approach to prevent biofilm formation consists in using biocides that have mostly been developed to target exponentially growing planktonic microorganisms, but these substances are poorly effective against biofilms [11]. Moreover, the toxic substances used as antifouling agents can be harmful to the natural environment [12]. Alternative preventive and curative approaches are currently being developed to specifically target mechanisms involved in biofilm formation or biofilm tolerance towards antimicrobials [7,11,13]. For example, enzymes inhibiting biofilm formation and disrupting pre-existing biofilms were shown to directly target the components of biofilm matrix by degrading the EPS [14,15]. Furthermore, this important field of investigation requires the development of ecofriendly anti-biofilm molecules [12,13,16,17]. Various studies have demonstrated that marine microbes are promising potential sources of bioactive compounds, including antibiofilm molecules, that act by regulating biofilm architecture, by inhibiting the attachment of microorganisms and thus the settlement of invertebrate larvae and macro-algal spores or by mediating the release of cells from biofilms during the dispersal stage of the biofilm life cycle [15,18,19,20,21,22,23].

The *Pseudoalteromonas* genus is predominant in the marine microbiome. These Gram-negative bacteria belong to the Gammaproteobacteria class, and are known to produce a variety of compounds of biotechnological interest, including anti-biofilm molecules [24,25]. Thus, anti-biofilm activities secreted by *Pseudoalteromonas* sp. 3J6 isolated from glass slides immersed in the Morbihan gulf (Brittany, France) [26] and *Pseudoalteromonas* sp. D41 isolated from a Teflon coupon immersed in the Bay of Brest (Brittany, France) [27] were characterized [18,28,29]. Likewise, the Antarctic marine bacterium *Pseudoalteromonas haloplanktis* TAC125, when grown with a sessile life-style, was shown to strongly inhibit the adhesion of *Staphylococcus epidermidis* [20,21,30]. Recently, an antibiofilm substance produced by *Pseudoalteromonas ruthenica* KLPp3 was identified as belonging to the diketopiperazine family [31] and purified alginate lyase (AlyP1400) produced by *Pseudoalteromonas* sp. 1400 was shown to disrupt the established biofilms of *Pseudomonas aeruginosa* [15].

In a previous study, we built up a collection of culturable marine bacteria isolated from corrosion product layers, which occurred during the early stages of marine corrosion of carbon steel [9] and screened it for the ability of the bacteria to form biofilms [32]. *Roseovarius* sp. VA014 strain was one of the interesting target models we selected because it develops stable biofilms on steel, polystyrene and glass surfaces [32]. Furthermore, members of Alphaproteobacteria (mainly *Roseovarius* and *Roseobacter* strains) are considered as pioneer surface colonizers, particularly on metallic surfaces [33]. The presence of *Roseovarius* sp. strains during early colonization events indicates that these bacteria could play an important role in the formation of marine biofilms by influencing the establishment of other colonizers in this environment.

In the current study, the marine *Pseudoalteromonas* sp. IIIA004 strain was identified as producing a strong anti-biofilm activity against *Roseovarius* sp. VA014. We showed that *Pseudoalteromonas* sp. IIIA004 exoproducts were particularly effective in disrupting *Roseovarius* sp. VA014 mature biofilms, but also in inhibiting an early stage of biofilm formation, the adhesion to substratum, without killing the bacteria or inhibiting their growth. A proteinaceous molecule, inhibiting the adhesion of *Roseovarius* sp. VA014, was purified and tested against a broad spectrum of bacteria, which demonstrated the promising potential of this novel molecule.

## 2. Materials and Methods

### 2.1. Bacterial Strains and Growth Conditions

Bacterial strains used in this study are listed in Table 1. The *Pseudoalteromonas* sp. IIIA004 strain producing anti-biofilm activity and the target *Roseovarius* sp. VA014 strain were isolated from the same habitat: corroded carbon steel coupons immersed in La Rochelle harbor (Atlantic coast, France) [9]. Marine isolates were grown in Zobell broth (pastone Bio-Rad, 4 g L^−1^; yeast extract Bio-Rad, 1 g L^−1^; sea salts Sigma-Aldrich, 30 g L^−1^) at 22 °C with shaking (150 rpm). Luria-Bertani broth (Difco) was used for the growth of non-marine strains at 37 °C with shaking (150 rpm). Solid media were prepared by adding agar (12 g L^−1^, Biokar). 

### 2.2. Preparation of the Pseudoalteromonas sp. IIIA004 Supernatant (SN_IIIA004_)

*Pseudoalteromonas* sp. IIIA004 was grown overnight at 22 °C in Zobell broth supplemented with 30 g L^−1^ of glucose with shaking for 48 h to optimize the production of antibiofilm compounds. The supernatant, named SN_IIIA004_, was harvested by centrifuging the culture (15 min, 7000× *g* at 4 °C), filter sterilized through 0.22 µm (Millipore PVDF), and stored at −80 °C until use. For some experiments, SN_IIIA004_ was concentrated 10-fold by lyophilization at a pressure below 450 mTorr at −80 °C (Cryotec freeze-dryer) and named 10X SN_IIIA004_.

### 2.3. Anti-Biofilm Assays

Microtiter plate assay (static conditions/polystyrene surface). Bacterial biofilms (marine and non-marine bacteria) were grown in microtiter plates as previously described by Doghri et al. [32]: an overnight bacterial culture was centrifuged 10 min at 7000 *g* and resuspended in artificial seawater (sea salts Sigma-Aldrich, 35 g L^−1^) for marine strains or in saline solution (NaCl 9 g L^−1^) for non-marine strains, to a final optical density at 600 nm (OD_600_) of 0.25. A total of 150 µl of the resulting suspensions were then loaded per well of a 96-well microtiter plates (MICROTEST^TM^ 96, Falcon). Artificial seawater or saline solution, without bacteria, served as negative controls. After a bacterial adhesion step of 2 h at 22 °C (marine bacteria) or 37 °C (non-marine bacteria), the wells were gently washed three times with artificial seawater or saline solution, respectively, and 150 µl of Zobell or LB medium, respectively, were added to each well. After incubation at 22 °C or 37 °C for 24 h, the microplates were washed three times. The bacterial biofilms were then stained with a 0.8% *w/v* crystal violet solution for 20 min and rinsed with ultra-pure water until the wash-liquid was clear. Crystal violet was then eluted from attached cells with 96% ethanol (150 µL per well) and the quantification was carried out by measuring the OD_595nm_. 

To investigate the effect of SN_IIIA004_ on bacterial adhesion and biofilm formation, wells were inoculated with biofilm-forming cells resuspended in a solution of 50% *v/v* SN_IIIA004_ and 50% *v/v* artificial seawater. After a 2 h adhesion step, biofilm formation was performed as described above. In this experiment, the culture supernatant was replaced with sterile culture medium in the negative control. Three independent experiments were performed, and for each experiment, the test was repeated in at least three wells per microtiter plate.

Flow cells assay (dynamic conditions/glass surface). *Roseovarius* sp. VA014 was grown on glass slides in three-channel flow cells (channel dimensions: 1 by 4 by 40 mm) (Technical University of Denmark Systems Biology), as previously described [32]: the flow cells were inoculated with overnight bacterial cultures diluted in artificial seawater to a final OD_600_ of 0.1. Bacteria were allowed to attach to the substratum (microscope glass coverslip of 24 × 50 st1, Knittel Glasser) during 2 h at 22 °C without a flow of medium. The channels were then washed to remove non-attached bacteria by applying a flow of artificial seawater for 15 min at a rate of 2 mL h^−1^ and biofilm growth was performed under a constant flow (2 mL h^−1^) of Zobell broth for 24 h at 22 °C. 

To investigate the effect of SN_IIIA004_ on adhesion and biofilm formation of *Roseovarius* sp. VA014, several protocols were followed. (i) A solution of 50% *v/v* SN_IIIA004_ and 50% *v/v* artificial seawater was injected without bacteria into the flow cell channels and left for 2 h at 22 °C without flow to coat the glass surface. The channels were then rinsed with artificial seawater before inoculating *Roseovarius* sp. VA014 bacteria and growing biofilms as described before. (ii) Flow cells were inoculated with *Roseovarius* sp. VA014 cultures grown for 24 h and resuspended in a solution of 50% *v/v* SN_IIIA004_ and 50% *v/v* artificial seawater to a final OD_600_ of 0.1. After the 2-h adhesion step, biofilm formation was performed as initially described. (iii) SN_IIIA004_ was injected into the channels after the *Roseovarius* sp. VA014 biofilm maturation step and left for 2 h under static conditions at 22 °C. For each experiment, the culture supernatant was replaced with sterile culture medium in the negative controls.

Microscopic observations were performed by confocal laser scanning microscopy (CLSM) using a TCS-SP2 system (Leica Microsystems, Mannheim, Germany). The biofilms formed were observed by staining the bacteria with 5 µM Syto 61 red for 10 min. The biofilm stacks were analyzed with the COMSTAT software (developed in MATLAB [40]) to estimate the maximal and average thicknesses (µm) and the biovolume (µm^3^ µm^−2^) of the biofilm. Each experiment was repeated three times, and three zones of each channel were analyzed per experiment.

### 2.4. Antibacterial Assays

Agar well diffusion assay. The effect of SN_IIIA004_ on the growth of *Roseovarius* sp. VA014 bacteria was assayed by adapting the agar well diffusion assay previously described by Sablé et al. [41]. Solid nutrient plates (15 mL) were inoculated with approximately 10^7^ cells of the target *Roseovarius* sp. VA014 strain. Sterile glass rings (4 mm inside diameter) were placed on agar medium and filled with 30 μL of filter-sterilized culture supernatant (SN_IIIA004_ or 10X SN_IIIA004_). The plates were incubated for 48 h at 22 °C, the optimum growth temperature for the target strain, to allow its growth and the culture supernatant diffusion. The presence of a halo around the glass cylinder indicates an inhibition of bacterial growth if the halo is clear (without cell growth) or eventually a stimulation if the halo is denser than the remaining plate. SN_IIIA004_ and 10X SN_IIIA004_ were replaced with Zobell broth and 10X Zobell broth in the respective negative controls.

Liquid antibacterial assay. Target *Roseovarius* sp. VA014 bacteria grown overnight were resuspended in a solution of 50% *v/v* SN_IIIA004_ and 50% *v/v* artificial seawater at an OD_600nm_ of 0.25 and incubated for 2 h. An aliquot of the cell suspension was then serially diluted and 100 µL of each dilution were plated, and the colony forming units (CFU) were counted after overnight growth. The remaining undiluted bacterial suspension was centrifuged, resuspended at 20% into fresh medium, and growth was monitored by measuring the absorbance of the cultures at 600 nm.

### 2.5. Microbial Adhesion to Solvent (MATS) Assays

The hydrophobic/hydrophilic and Lewis acid-base characteristics of the *Roseovarius* sp. VA014 surface were determined using the MATS method described by Bellon-Fontaine et al. [42]. This partitioning method is based on the comparison between microbial cell affinity to couples of solvents. In each pair, one solvent is a monopolar solvent, the other is an apolar solvent, and both must have similar Lifshitz-van der Waals surface tension components. The monopolar solvent can be acidic (electron accepting) or basic (electron donating). The following couples were used: (i) ethyl acetate (electron donating)/decane; (ii) dichloromethane (electron accepting)/tetradecane. All solvents were obtained from Sigma-Aldrich and were of the highest purity grade. Differences between the results obtained with dichloromethane and tetradecane, on the one hand, and between ethyl acetate and decane, on the other hand, indicate the electron donor and the electron acceptor character, respectively, of the bacterial surface. The percentage of cells adhered to tetradecane was used as a measure of cell surface hydrophobicity. Experimentally, a *Roseovarius* sp. VA014 suspension, containing approximately 10^8^ cells mL^−1^ (OD_400 nm_ = 0.8), was prepared in artificial seawater. Moreover, 1.5 mL of bacterial suspension was manually mixed for 10 s and vortexed for 120 s with 0.25 mL of the solvent under investigation. The mixture was allowed to stand for 15 min to ensure complete separation of the two phases. The solvent phase was carefully removed and the OD of the aqueous phase was measured at 400 nm. The percentage affinity of bacteria to each solvent was calculated by % Affinity = (1−A/A_0_) × 100, where A_0_ is the OD_400 nm_ of the bacterial suspension before mixing and A is the OD_400 nm_ after mixing.

### 2.6. Physico-Chemical Characterization of the Active Compound(s)

To elucidate the biochemical nature of the active compound(s), different treatments were performed on SN_IIIA004_. Proteinase K or pronase E were added to SN_IIIA004_ at a final concentration of 1 mg mL^−1^ to digest proteins and the reaction mixture was incubated for 1 h at 37 °C. To degrade lipids, lipase acrylic resin form was used at a final concentration of 2 mg mL^−1^ and the reaction was incubated for 48 h under shaking at 37 °C. DNaseI (100 µg mL^−1^) or RNaseA (25 µg mL^−1^) was added for 12 h at 37 °C to digest the nucleic acids. NaIO_4_ was used at a final concentration of 20 mM to hydrolyze polysaccharides by cleaving the C-C bonds and by oxidizing the carbon of vicinal hydroxyl groups [43,44]. After an incubation for 2 h at 37 °C, the excess of NaIO_4_ was neutralized with ethylene glycol (1:100) [45] for 2 h at 37 °C. This step was followed by a final overnight dialysis (molecular weight cut-off: 1000 Da, Spectrum Labs.com). To evaluate the heat sensibility, SN_IIIA004_ was incubated for 1 h at 37 °C, 30 min at 50 °C, 70 °C, or 100 °C. SN_IIIA004_ was replaced with Zobell broth in the negative controls.

After each of the above treatments, the resulting SN_IIIA004_ was assayed for anti-biofilm activity by using the microtiter plate assay as described above and its activity was compared with that of the untreated SN_IIIA004_.

### 2.7. Biosurfactant Assay

In order to detect the presence of biosurfactant in SN_IIIA004_, we used the drop-collapse test as described by Tugrul and Cansunar [46]. Briefly, drops of SN_IIIA004_ were placed in wells of a microtiter plate coated with sunflower oil. Drops containing biosurfactant would collapse whereas surfactant-free drops would remain stable. Diluted liquid hand washing cream, distilled water and Zobell broth drops were used as controls. 

### 2.8. Purification of the SN_IIIA004_ Anti-Biofilm Compound 

A two-step purification protocol was used. In the first step, the crude supernatant was applied to a HyperSep™ C_18_ solid phase extraction column (500 mg, 6 mL, Thermo Scientific). Five stepwise elutions were successively performed with 100% Milli-Q water, 20%, 40%, 60% acetonitrile in Milli-Q water and 100% acetonitrile. After acetonitrile evaporation, the different fractions were tested for anti-biofilm activity towards *Roseovarius* sp. VA014 by using the microtiter plate assay as described above.

The 20% acetonitrile active fraction was concentrated by lyophilization and resuspension in Milli-Q water, filtered, and subjected to reverse-phase-high-performance liquid chromatography (RP-HPLC). This second purification step was performed using a C_18_ column (3.5 µm, 150 × 4.6 mm, XSELECT CSH 130, Waters) and conducted with a Waters system (600 Controller, 2996 Photodiode Array detector and 2707 Autosampler). Separation was performed with the following acetonitrile gradient in Milli-Q water: 0 to 20% for 20 min, 20 to 50% for 2 min, 50 to 60% for 2 min, 60 to 90% for 2 min, at a flow rate of 1 mL min^−1^. Each eluted fraction, collected according to the chromatographic profile obtained at 215 nm, was concentrated by lyophilization and resuspension in Milli-Q water. For each fraction, the protein concentration was determined by the bicinchoninic acid method (BC protein assay kit, Sigma-Aldrich), using bovine serum albumin as standard and the anti-biofilm activity was tested by using the microtiter plate assay as described above. 

The purified active fraction was then subjected to an additional RP-HPLC analysis (the applied gradient is shown on Figure 6b). All the collected fractions containing the pure active molecule were pooled, concentrated by lyophilization, and stored at −80 °C.

To evaluate the molecular weight of the anti-biofilm molecule, the active purified fraction was transferred into a Centricon tube (Millipore) with a 3 kDa Nominal Molecular Weight Limit (NMWL) and centrifuged at 7500× *g* for 40 min at 4 °C.

### 2.9. Statistical Analyses

All values presented in the “Results” section are the averages of three independent experiments. The standard deviations were calculated using MATLAB software (MathWorks Inc., Natick, MA, USA). For each experiment, at least three technical replicates were performed. In order to analyze differences between a sample and the corresponding control, Student’s t tests were performed. Differences were considered significant if *p* values were <0.05.

## 3. Results

### 3.1. Pseudoalteromonas sp. IIIA004 Exoproducts Inhibit Biofilm Formation by Roseovarius sp. VA014

**Figure 1:**
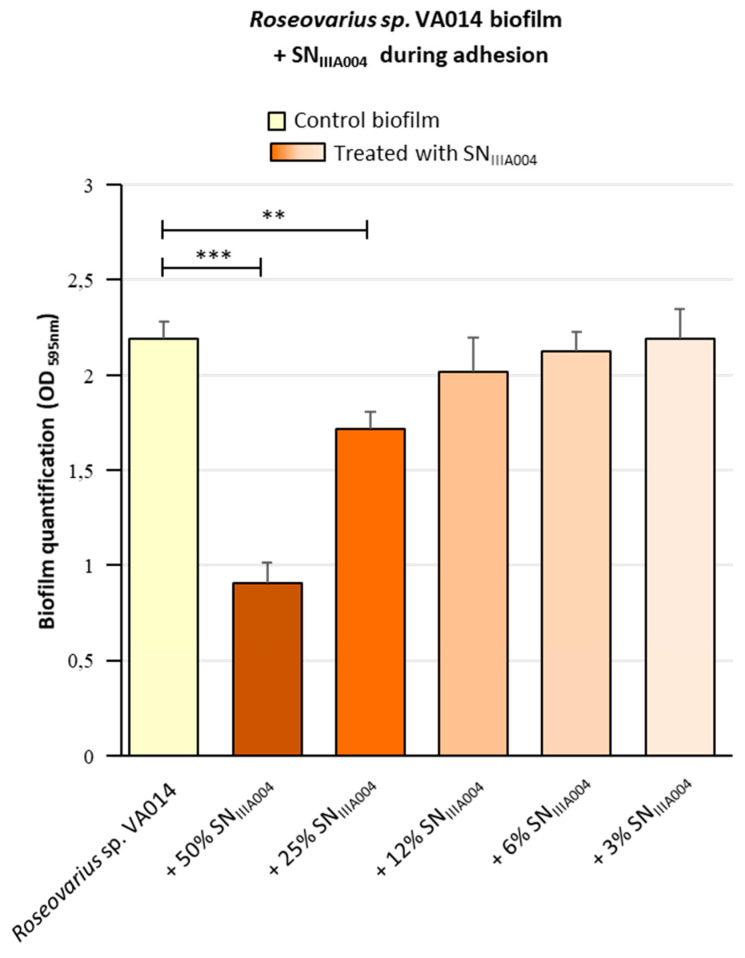
Effect of SN_IIIA004_ on the *Roseovarius* sp. VA014 biofilm formation in microtiter plate. *Roseovarius* sp. VA014 was mixed for 2 h with serial dilutions of SN_IIIA004_ in 96-well microplates during the adhesion step. The *Roseovarius* sp. VA014 biofilms were then grown at 22 °C for 24 h. The standard deviations were calculated from 3 replicates. Control biofilm: *Roseovarius* sp. VA014 biofilm treated with Zobell broth instead of SN_IIIA004_. Each biofilm treated with a culture supernatant was compared with the control biofilm. Significant differences are indicated by ** (*p* < 0.01) or *** (*p* < 0.001).

**Figure 2:**
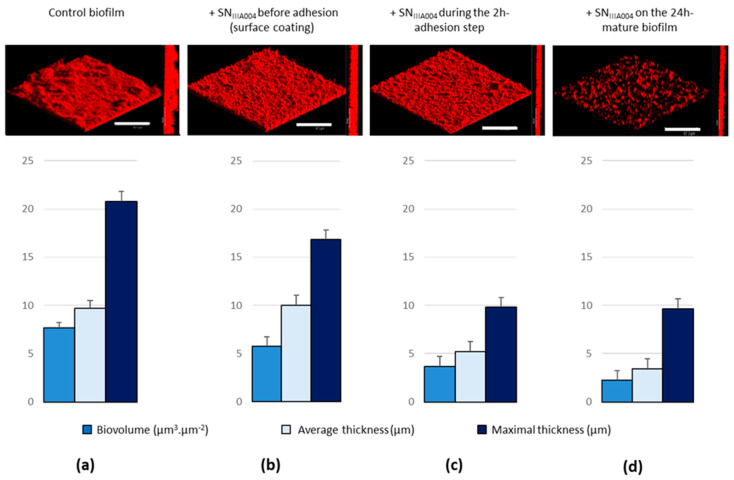
Effect of SN_IIIA004_ on the *Roseovarius* sp. VA014 biofilm formation in flow cell chambers. The *Roseovarius* sp. VA014 biofilms were grown at 22 °C for 24 h in Zobell broth after the 2 h adhesion step. (**a**) Control biofilm: *Roseovarius* sp. VA014 biofilm treated with Zobell broth instead of SN_IIIA004_. (**b**) SN_IIIA004_ was added before the bacteria, to coat the glass surface for 2 h. (**c**) SN_IIIA004_ was added together with the bacteria during the 2 h adhesion step. (**d**) SN_IIIA004_ was added after biofilm maturation and incubated for 2 h. For each experiment, a three-dimensional (3D) representation and a side view projection are shown. Average/maximal thicknesses and biovolumes were calculated, for each experiment, from COMSTAT analyses of 10 images stacks obtained from two independent biofilms. The standard deviations were lower than 10% of each value. White bars = 67.3 µm.

The *Pseudoalteromonas* sp. IIIA004 marine bacterium was isolated from a complex biofilm closely linked with corrosion products formed on carbon steel structures immersed in a French Atlantic harbor [9]. Here, we found that the culture supernatant of this strain, SN_IIIA004_, inhibited the biofilm formation of another bacterial strain sharing the same habitat, *Roseovarius* sp. VA014 (Figure 1 and Figure 2). When we examined the effect of increasing SN_IIIA004_ concentrations on biofilm formation in 96-well microtiter plate wells, the *Pseudoalteromonas* sp. IIIA004 secretome showed a dose-dependent anti-biofilm effect (Figure 1).

A reduction of about 50% of the *Roseovarius* sp. VA014 biofilm formation was observed at a 1:2 dilution (50% *v/v* SN_IIIA004_). This concentration was then used for all subsequent experiments. 

Although the polystyrene microtiter plate assay is a simple means of testing bacterial biofilm inhibition, it measures biofilm formation in static cultures, far from a naturally hydrodynamic environment. Therefore, the SN_IIIA004_ activity was further studied in a flow cell model (on glass surface) that allowed the continuous flow of fresh nutrients into a chamber. The *Roseovarius* sp. VA014 strain was grown in flow cell in the absence or presence of SN_IIIA004_ and biofilms were subsequently analyzed using confocal laser scanning microscopy (Figure 2).

The addition of SN_IIIA004_ before the *Roseovarius* sp. VA014 strain (glass-coating with SN_IIIA004_, Figure 2b) did not significantly prevent the biofilm formation, which demonstrated that SN_IIIA004_ is devoid of components able to act on the abiotic surface to reduce *Roseovarius* sp. VA014 adhesion. On the contrary, when added during the 2 h adhesion step, SN_IIIA004_ halved the biofilm formation of *Roseovarius* sp. VA014 (Figure 2c), as observed in static conditions in microtiter plates (Figure 1). These findings demonstrated that SN_IIIA004_ also had biofilm inhibitory activity in flow cell under dynamic conditions and that the differences in surface nature (glass or polystyrene) did not influence the anti-biofilm activity. 

### 3.2. SN_IIIA004_ Disrupts the Established Bacterial Biofilm

While SN_IIIA004_ exhibited an inhibitory activity against bacterial biofilm formation, it was of interest to explore whether the established biofilms were also sensitive to *Pseudoalteromonas* sp. IIIA004 exoproducts. This assay was performed by growing mature biofilms of the target strain in flow cell chambers before adding SN_IIIA004_. The results showed a significant reduction of thicknesses and biovolumes of *Roseovarius* sp. VA014 biofilms (Figure 2d): about 60% of mature biofilm disappeared. These findings demonstrated that SN_IIIA004_ contained components able to modify the properties of preformed biofilms and/or to destroy them.

### 3.3. SN_IIIA004_ is Devoid of Bactericidal Activity against Free-Living Cells 

**Figure 3:**
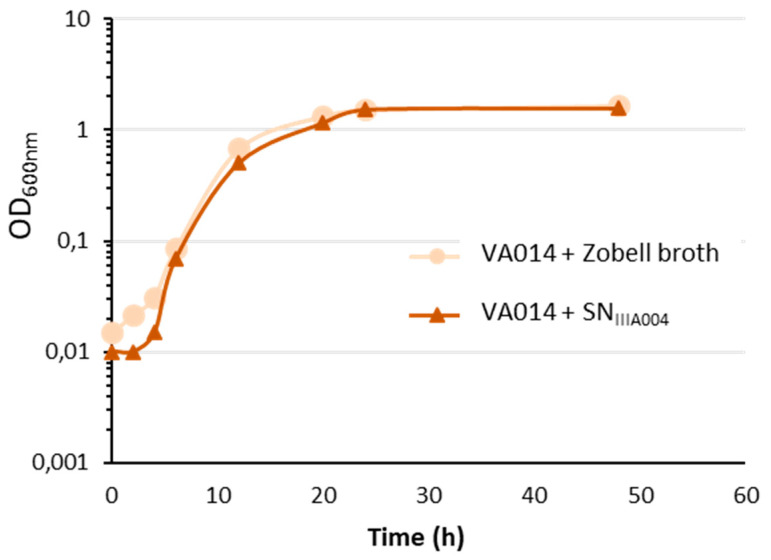
Growth curves of *Roseovarius* sp. VA014 pre-incubated with Zobell broth (control) or SN_IIIA004_ for 2 h. The growth was monitored by measuring the OD_600nm_.

Since SN_IIIA004_ inhibited biofilm formation and disrupted preformed biofilms, we examined whether SN_IIIA004_ contained an antibacterial substance that could be responsible for these effects. Using the agar well diffusion assay, neither crude SN_IIIA004_ nor concentrated 10X SN_IIIA004_ inhibited the growth of *Roseovarius* sp. VA014, indicating that SN_IIIA004_ was neither bactericidal nor bacteriostatic toward this target strain. The number of CFU mL^−1^ was evaluated after incubation of *Roseovarius* sp. VA014 for 2 h with SN_IIIA004_ or with Zobell broth (control), and was similar in both cases (6 × 10^8^ CFU mL^−1^). Moreover, the number of bacteria was the same for both conditions throughout the experiment (Figure 3). 

These results demonstrated that the viability of planktonic bacteria was not affected by the *Pseudoalteromonas* sp. IIIA004 exoproducts and suggested that SN_IIIA004_ did not reduce cell viability during the 2 h-adhesion step and the subsequent biofilm formation stages.

### 3.4. SN_IIIA004_ Does Not Change the Hydrophilic and Acid Character of the Roseovarius sp. VA014 Cell Surface 

**Figure 4:**
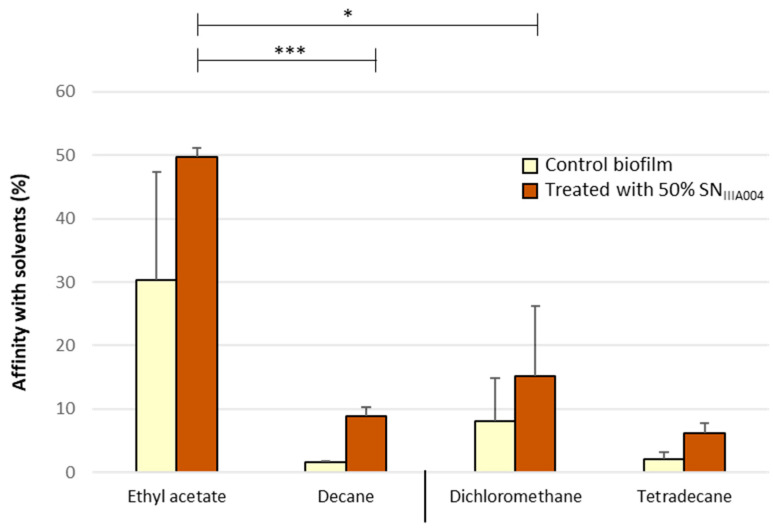
Percentage affinity of *Roseovarius* sp. VA014 cells to the four solvents used in the Microbial Adhesion To Solvents (MATS) method. *Roseovarius* sp. VA014 cells were pretreated with SN_IIIA004_ or Zobell broth (control). Two couples of solvents were used: ethyl acetate (monopolar basic)/decane (apolar) and dichloromethane (monopolar acidic)/tetradecane (apolar). Percentage affinities are mean values (±SD) of six experiments obtained from at least two independent treatments. Affinity differences are indicated by * (*p* < 0.05) or *** (*p* < 0.001).

Another hypothesis was that SN_IIIA004_ could modify the properties of bacterial surface and thus affected cell adhesion. We used the Microbial Adhesion To Solvents (MATS) method to determine the hydrophobic/hydrophilic and Lewis acid/base characteristics of *Roseovarius* sp. VA014 surface, incubated with SN_IIIA004_ or Zobell broth only (for the control) for 2 h. This partitioning method is based on the comparison of bacterial cell affinity to a monopolar acidic (electron acceptor) or basic (electron donator) solvent and an apolar solvent that have similar Lifshitz-van der Waals surface tension components. The solvent percentage affinity of cells to each solvent is shown in Figure 4. 

Whatever the treatment (Zobell broth or SN_IIIA004_), the *Roseovarius* sp. VA014 cell surfaces presented a hydrophilic character by showing a very low affinity for the apolar solvents (decane, tetradecane). Moreover, the percentage affinity for the basic polar solvent (ethyl acetate) was much higher than for dichloromethane, indicating that the cell surface presented a broadly acidic character, whether treated with Zobell broth or with SN_IIIA004_. 

### 3.5. Physico-Chemical Characteristics of SN_IIIA004_ Anti-Biofilm Compounds

**Figure 5:**
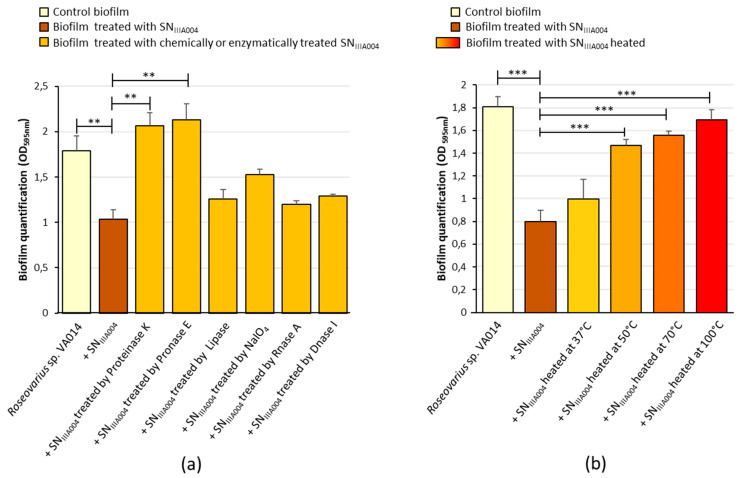
Effect of various treatments on the anti-biofilm activity of SN_IIIA004_. Microtiter plate assay was used. (**a**) Effect of the digestion of proteins (proteinase K, pronase E), lipids (lipase), polysaccharides (NaIO_4_), and nucleic acids (RNaseA, DNaseI) on the anti-biofilm activity of SN_IIIA004_. (**b**) Effect of increasing heat treatments on the anti-biofilm activity of SN_IIIA004_. Control biofilm: *Roseovarius* sp. VA014 biofilm treated with Zobell broth instead of SN_IIIA004_. The data represent mean values ± standard deviations of at least three replicates. The effect of each SN_IIIA004_ submitted to a chemical or thermal treatment was compared with the effect of the native SN_IIIA004_. Significant differences are indicated by ** (*p* < 0.01) or *** (*p* < 0.001).

To gain information on the biochemical nature of the active compounds, we examined whether the anti-biofilm molecules present in the *Pseudoalteromonas* sp. IIIA004 secretome retained their activity after different treatments (Figure 5). The only treatments that completely canceled the antibiofilm activity of SN_IIIA004_ were treatments with proteinase K and pronase E. This finding was made both under static (Figure 5a) and dynamic (data not shown) conditions. The SN_IIIA004_ anti-biofilm compounds were also heat-sensitive (Figure 5b): the inhibitory effect was significantly affected from 50 °C and clearly decreased when further increasing the temperature.

This clearly demonstrated the proteinaceous nature of the active compounds. Moreover, SN_IIIA004_ drops did not collapse and remained stable on oil-coated surface, showing that SN_IIIA004_ is devoid of biosurfactant activity.

### 3.6. Isolation and Purification of the Anti-Biofilm Molecule

**Figure 6:**
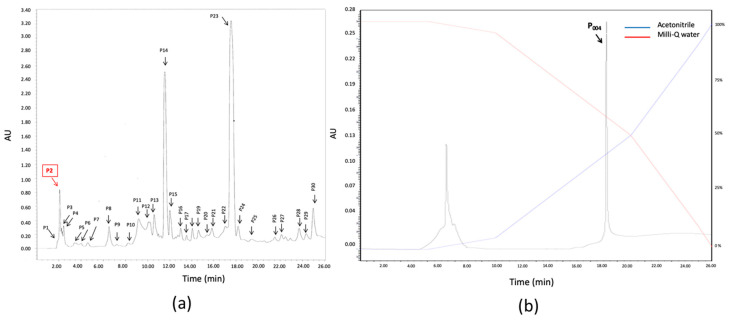
Reverse-phase-high-performance liquid chromatography (RP-HPLC) elution profile of the SN_IIIA004_ active fraction (**a**) chromatographic profile of the 20% acetonitrile anti-biofilm fraction eluted by solid-phase extraction on HyperSep C_18_ cartridges of SN_IIIA004_. Only one fraction, P2 (2nd pic), presented anti-biofilm activity. (**b**) Chromatographic profile of the P2 fraction eluted by the first RP-HPLC separation. Eluents were Milli-Q water and acetonitrile.

The proteinaceous molecule responsible of the anti-biofilm activity was purified to homogeneity from the stationary-phase culture supernatant of the *Pseudoalteromonas* sp. IIIA004 producer. The 20% acetonitrile fraction (50 µg of proteins), obtained by solid-phase extraction on HyperSep C_18_ cartridges, showed a specifically anti-biofilm activity against *Roseovarius* sp. VA014. This fraction was concentrated by lyophilization and then subjected to an accurate separation by two successive C_18_ RP-HPLC. After each separation, only one fraction, corresponding to one OD peak (P2, 10 µg of proteins, Figure 6a and P_004_, 3 µg of proteins, Figure 6b), presented anti-biofilm activity, showing that no other anti-biofilm molecule was co-purified. From the final C_18_ RP column, the active molecule was eluted in a single peak at 40 % of acetonitrile. 

**Figure 7:**
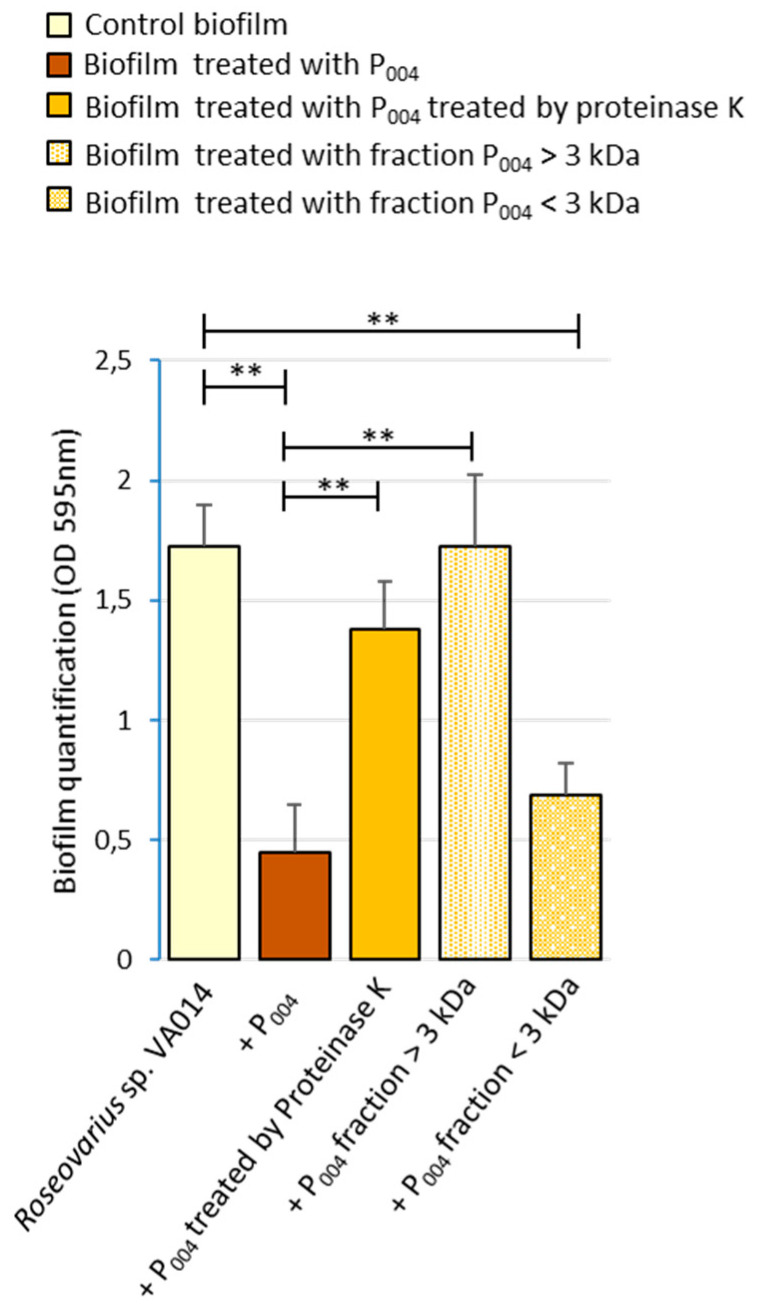
Effect of proteinase K treatment on the P_004_ anti-biofilm compound purified from SN_IIIA004_ and molecular weight evaluation. Microtiter plate assay was used. P_004_ was either treated with proteinase K or filtered in a Centricon tube with a 3 kDa Nominal Molecular Weight Limit (NMWL) (>3 kDa: retentate; <3 kDa: filtrate). Control biofilm: *Roseovarius* sp. VA014 biofilm treated with Zobell broth instead of SN_IIIA004_. Significant differences are indicated by ** (*p* < 0.01).

The anti-biofilm activity of the HPLC purified eluate treated by proteinase K was also highly affected (70% of loss, Figure 7). This confirmed the proteinaceous nature of the molecule responsible for the anti-biofilm activity. 

The purified eluate was submitted to a centrifugal Centricon filter that retains the components with a molecular weight higher than 3 kDa. The anti-biofilm activity was detected in the filtrate only (Figure 7), clearly showing that the molecular weight of the active component was lower than 3 kDa. This pure molecule was named P_004_.

### 3.7. Spectrum of Action of the P_004_ Anti-Biofilm Molecule Purified from SN_IIIA004_

The microtiter plate assay was used to determine the spectrum of action of the pure compound. P_004_ (10 µg) was assayed against 21 bacterial strains able to form stable biofilms in microtiter plates with an OD_595nm_ > 1 after crystal violet staining. These bacteria include human pathogens, pathogenic marine bacteria such as *Flavobacterium*, *Tenacibaculum,* and *Vibrio lentus*, as well as bacteria potentially involved in biocorrosion or biofouling in the marine environment (Table 1). The percentages of inhibition of P_004_ on monospecies biofilms are presented in Table 2. 

The most sensitive strains, with inhibition percentages ranging from 62 to 81.5%, were both bacteria widely distributed in the marine environment (*Roseovarius* sp., *Paracoccus* sp., and *Micrococcus luteus*) and well-known human pathogens (*Staphylococcus aureus*, *Yersinia enterocolitica,* and *Pseudomonas aeruginosa*). A second group of bacteria, less sensitive (inhibition percentages from 12 to 45%), included another strain of *S. aureus*, two *Bacillus* strains and several marine bacteria belonging to the *Flavobacteriaceae* family (*Zobellia galactanivorans, Tenacibaculum* sp., *Flavobacterium* sp., and *Cellulophaga lytica*). Some strains were not sensitive to the P_004_ biofilm-inhibiting molecule (inhibition percentage < 10%), especially marine bacteria of the *Vibrio* genus and one *Pseudomonas aeruginosa* strain.

## 4. Discussion

This study reports the isolation and characterization of a new anti-biofilm compound from the *Pseudoalteromonas* sp. IIIA004 marine bacterium, active against various bacteria. As described in several studies, marine bacteria are a potential source of effective anti-biofilm compounds [23]. Some of them were shown to secrete anti-biofilm molecules active against a wide range of Gram-positive and Gram-negative bacteria including *Staphylococcus aureus*, *Listeria monocytogenes,* and *Salmonella typhimurium* [47]. Here, we examined the in vitro activity of the cell-free supernatant of *Pseudoalteromonas* sp. IIIA004 against the *Roseovarius* sp. VA014 marine strain, identified among the pioneer and sustaining surface colonizers particularly on metallic surfaces [9]. This anti-biofilm activity was characterized in microtiter plates (static conditions/polystyrene surface) and in flow cell chambers (dynamic conditions/glass surface). Both techniques highlighted the anti-biofilm potential of this strain. SN_IIIA004_ was shown to display its activity during two stages of biofilm formation: (i) when the culture supernatant was mixed with *Roseovarius* sp. VA014 cells during the 2 h adhesion step, biofilm growth was inhibited both on polystyrene and glass surfaces, and (ii) when the culture supernatant was added after the *Roseovarius* sp. VA014 biofilm formation, the mature biofilm was strongly dispersed. The anti-biofilm effect of *Pseudoalteromonas* sp. IIIA004 exoproducts is particularly interesting because it combines several activities, as previously demonstrated for the marine bacterial exopolysaccharide EPS273 that exhibited an inhibition of *Pseudomonas aeruginosa* PAO1 biofilm formation as well as a biofilm dispersion [48]. From 2001, disturbing the multicellular structure of bacterial biofilm was suggested as a promising way to increase antibiotic sensitivity of pathogens in biofilms [49]. Recently, innovative strategies combining antibiotics and anti-biofilm compounds such as polysaccharides [50], synthetic peptides [51], or alginolytic enzymes [15] were proposed in order to increase the susceptibility of microbial biofilms to antibiotics. Interestingly, antibacterial assays with SN_IIIA004_ demonstrated the lack of bactericidal or bacteriostatic action against free-living *Roseovarius* sp. VA014 cells. Therefore, the anti-biofilm activity of *Pseudoalteromonas* sp. IIIA004 is likely to be mediated by mechanisms different from growth inhibition. By contrast, most of the known anti-biofilm molecules are bactericidal or bacteriostatic, such as for instance the AlpP protein secreted by *Pseudoalteromonas tunicata* [52] or the bioactive compounds of *Pseudoalteromonas* sp. IBRL PD4.8 [53]. The main characteristics of various anti-biofilm molecules produced by different *Pseudoalteromonas* strains are summarized in Table 3.

Among the few natural molecules displaying anti-biofilm activity without affecting cell viability, some polysaccharides [47,54] and biosurfactants [55] are well known. We showed that the SN_IIIA004_ anti-biofilm compound was neither a polysaccharide nor a biosurfactant. We further investigated whether this compound could act by modifying the properties of bacterial cells and/or abiotic surfaces. MATS results clearly showed that SN_IIIA004_ did not significantly affect the hydrophilic and acid properties of the *Roseovarius* sp. VA014 cell surface. Moreover, no glass-coating effect was observed with SN_IIIA004_ compounds. All these findings suggested that the SN_IIIA004_ anti-biofilm compound did not act on surfaces, whether or not biotic. Other possible mode of action could be considered. Since SN_IIIA004_ anti-biofilm action is effective during cell adhesion and on mature biofilm without affecting cell multiplication, the anti-biofilm compounds could act as signaling molecules that modulate gene expression of target bacteria, as suggested for several anti-biofilm polysaccharides [47]. For instance, exopolysaccharides released from *Lactobacillus acidophilus* A4 down-regulated several *E. coli* genes related to adhesive properties (curli genes) [56]. Moreover, *Pseudoalteromonas* sp. IIIA004 exoproducts might block lectins or adhesins of fimbriae and pili on the surface of bacteria, which could interfere with the cell-surface and cell-cell adherence, as suggested for the marine bacterial exopolysaccharide A101 [54] or for some microbial branched polysaccharides used as food additives [57]. However, as P_004_ does not affect the viability of the tested bacteria, it certainly does not act on membrane permeability, unlike some antibacterial molecules [58].

Among the major kinds of molecules that impair biofilm maturation, there are also the quorum-sensing (QS) inhibitors [7,11,17]. Some enzymes such as *N*-acyl homoserine lactone (AHL)-lactonases and AHL-acylases degrade signal QS molecules and thus prevent the cell-to-cell communication, which in turn impairs population behavior such as biofilm development [59,60]. Other kind of enzymes can directly target the exopolymeric matrix by degrading its components such as polysaccharides or extracellular DNA. Thus, the use of DNaseI [61], alpha-amylase [62] or Dispersin B [63] has been identified as an efficient means of dispersing biofilms, in vitro and in vivo. Investigation of the potential binding of the *Pseudoalteromonas* sp. IIIA004 purified exoproducts with adhesins or other matrix compounds would be necessary to better understand the mechanisms underlying their anti-biofilm effects.

The anti-biofilm effect of SN_IIIA004_ during the initial attachment step is likely due to a new molecule. Protease and heat treatments impaired SN_IIIA004_ ability to inhibit biofilm formation, indicating that the active molecule was of proteinaceous nature. The anti-biofilm peptide P_004_ was then purified from *Pseudoalteromonas* sp. IIIA004 exoproducts and its molecular weight was estimated to less than 3 kDa. To our knowledge, no bacterial molecule with both this molecular weight and a specific anti-biofilm activity (without affecting bacterial viability) has been described yet. On the contrary, several small peptides (natural or chemically synthesized) with dual antimicrobial and anti-biofilm activity have been reported [66,67]. Moreover, the *Pseudoalteromonas* genus is of great interest to the scientific community because of its prolific metabolite-producing ability [25,68]. Compounds of interest include toxic proteins, polyanionic exopolymers, substituted phenolic and pyrrole-containing alkaloids, cyclic peptides and a range of bromine-substituted compounds with antimicrobial, anti-fouling, algicidal, and various pharmaceutically relevant activities [23,25]. However, among the anti-biofilm proteinaceous compounds synthesized by *Pseudoalteromonas* strains, only the 190-kDa autotoxic protein (AlpP) produced by *P. tunicata* D2 and the 23-kDa alginate lyase (AlyP1400) produced by *Pseudoalteromonas* sp. 1400 were purified and characterized (Table 3) [15,52,69]. *Pseudoalteromonas* sp. 3J6 and D41 were also identified as producing proteinaceous molecules with a specifically anti-biofilm activity against a wide range of various bacterial biofilms (Table 3) [18,28,29]. However, the anti-biofilm molecule from SN_3J6_ was eluted with 50% acetonitrile from a Sep-Pak Plus C_18_ cartridge and was recently identified as a 13-kDa protein named alterocin [65], while the SN_D41_ anti-biofilm molecule was unable to be eluted from the same column either with acetonitrile or with other solvents. These findings support the hypothesis that the anti-biofilm molecules of SN_3J6_ and SN_D41_ are different from the anti-biofilm molecule P_004_ eluted with 20% acetonitrile from a C_18_ cartridge. 

Finally, the new proteinaceous small molecule P_004_ presents a wide spectrum of action on various bacteria, with 71% of sensitive strains, including the human pathogens *Staphylococcus* aureus, *Pseudomonas aeruginosa* PAO1, and *Yersinia enterocolitica* (Table 3). Therefore, the potential use of P_004_ is not limited to the marine environment to inhibit undesirable bacteria in aquaculture, biofilms involved in biocorrosion or biofouling, but it could also be extended to the medical field.

## Figures and Tables

**Table 1:** Strains used in this study.

Strain	Reference and/or Source	Culture Conditions
**Non Marine Strains**		
*Staphylococcus aureus* AH478	[34]	Luria-Bertani 37 °C
*S. aureus* ATCC27217	ATCC	
*S. aureus* RN4220	[35]	
*Pseudomonas aeruginosa* PAO1	[36]	
*P. aeruginosa* PA14	[37]	
*Yersiniaenterocolitica* CIP106.676	CIP	
*Bacillus subtilis* ND Food	[38]	
*Bacillus thuringiensis* 407	[39]	
**Marine Strains**		
*Paracoccus* sp. 4M6	[26]/Morbihan Gulf, France	Zobell 22 °C
*Micrococcus luteus*	LBCM	
*Zobellia galactanivorans*	LBCM	
*Cellulophaga lytica* DSM2039	DSMZ	
*Cellulophaga lytica* DSM2040	DSMZ	
*Vibrio lentus* CIP107166T	CIP	
*Vibrio anguillarum* CIP6336T	CIP	
*Vibrio* sp. D01	[27]/Bay of Brest, France	
*Pseudoalteromonas* sp. IIIA004	[33]/Atlantic harbor, France	
*Roseovarius* sp. VA014	[9]/Atlantic harbor, France	
*Roseobacter* sp. IV 3009	[32]/Intertidal mudflat, France	
*Shewanella* sp. IV 3014	[32]/Intertidal mudflat, France	
*Flavobacterium* sp. II2003	[32]/Intertidal mudflat, France	
*Tenacibaculum* sp. II2021	[32]/Intertidal mudflat, France	

ATCC: American Type Culture Collection; CIP: Institut Pasteur Collection; DSMZ: Deutsche Sammlung von Mikroorganismen und Zellkulturen collection; LBCM: laboratory LBCM collection (Université de Bretagne-Sud, France).

**Table 2:** Spectrum of action of the P_004_ anti-biofilm molecule purified from SN_IIIA004_.

	P_004_ (10 µg)
Strain	Anti-biofilm Assays ^a^
*High inhibition level ^b^*	
*Roseovarius* sp. VA014	71.4 ± 2.2
*Staphylococcus aureus* AH478	71.4 ± 2.2
*Staphylococcus aureus* RN4220	62.2 ± 1.3
*Yersiniaenterocolitica* CIP106.676	81.5 ± 5.8
*Paracoccus* sp. 4M6	77.7 ± 1.9
*Pseudomonas aeruginosa* PAO1	80.9 ± 2.4
*Micrococcus luteus*	71.7 ± 8.3
*Mild Inhibition Level ^b^*	
*Staphylococcus aureus* ATCC27217	45.1 ± 5.3
*Zobellia galactanivorans*	42.7 ± 0.8
*Tenacibaculum* sp. II2021	40.2 ± 5.2
*Flavobacterium* sp. II2003	32.5 ± 1.7
*Cellulophaga lytica* DSM2039	26.3 ± 3.8
*Cellulophaga lytica* DSM2040	19.8 ± 2.1
*Bacillus thuringiensis* 407	17.9 ± 1.8
*Bacillus subtilis* ND Food	12.3 ± 0.9
*Non Sensitive Strains ^b^*	
*Pseudomonas aeruginosa* PA14	−0.38 ± 0.02
*Shewanella* sp. IV3014	−1.9 ± 0.2
*Roseobacter* sp. IV3009	−21.1 ± 1.1
*Vibrio anguillarum* CIP6336T	5.2 ± 0.7
*Vibrio lentus* CIP107166T	5.8 ± 0.4
*Vibrio* sp. D01	−12.1 ± 0.2

^a^ Inhibition percentage of biofilm formation in the presence of P_004_ with microtiter plate assay ± SD. ^b^ Groups distinguished on the basis of the inhibition percentage of biofilm formation in the presence of P_004_.

**Table 3:** Anti-biofilm compounds produced by *Pseudoalteromonas* sp. Bacteria.

Producing Bacterium	Source	Target Biofilms	Active Compounds	Action	References
***Pseudoalteromonas ulvae* TC14**	Mediterranean Sea, Bay of Toulon, France	*Persicivirga (Nonlabens) mediterranea, Shewanella* sp., *Alteromonas genovensis, Pseudoalteromonas* sp.	PS I and/or PS II (exo polysaccharides)	Inhibition of biofilm formation	[64]
***Pseudoalteromonas haloplanktis* TAC125**	Antarctic sea water	*Staphylococcus epidermidis*	Pentadecanal	- Inhibition of initial attachment - Modulation of the AI-2 quorum sensing system	[20,21,30]
***Pseudoalteromonas* sp. 3J6**	Glass slides immersed in the Morbihan gulf, France	*Vibrio* sp., *Pseudomonas aeruginosa, Escherichia coli, Salmonella enterica, Colwellia* sp., *Algibacter* sp., *Micrococcus* sp., *Paracoccus* sp.	13-kDa protein (Alterocin)	- Inhibition of initial attachment (*Vibrio tapetis*) - Inhibition of biofilm formation	[18,26,28,29,65]
***Pseudoalteromonas* sp. D41**	Teflon coupon immersed in the Bay of Brest, France	*Pseudoalteromonas* sp., *Paracoccus* sp.	Proteinaceous molecule	Inhibition of biofilm formation	[18,27]
***Pseudoalteromonasruthenica* KLPp3**	Marine crab in Pulau Perhentian, Malaysia	*Vibrio alginolyticus, Serratia marcescens*	Cyclic peptide of the diketopiperazine family	Inhibition of initial attachment and biofilm formation	[31]
***Pseudoalteromonas* sp. 1400**	Sea water of Queensland Beach, Canada	*Pseudomonas aeruginosa*	23-kDa alginate lyase (AlyP1400)	Disruption of the established biofilms	[15]
***Pseudoalteromonas tunicata***	Tunicates	*Pseudoalteromonas tunicata*	190-kDa autotoxic protein (AlpP)	Killing and detachment of the biofilm from the substratum	[52]
***Pseudoalteromonas* sp. IBRL PD4.8**	Green macroalgae (*Caulerpa racemose*) Port Dickson, Malaysia	*Vibrio alginolyticus*	Crude extracts	- Inhibition of the initial and pre-formed biofilms - Antibacterial activity against fouling bacteria	[53]
***Pseudoalteromonas* sp. IIIA004**	Corroded carbon steel coupons immersed in La Rochelle harbor, Atlantic coast, France	*Roseovarius* sp., *Staphylococcus aureus, Yersinia enterocolitica, Paracoccus* sp., *Pseudomonas aeruginosa* PAO1, *Micrococcus luteus, Flavobacterium* sp., *Tenacibaculum* sp., *Cellulophaga lytica*	- Proteinaceous molecule P_004_ - Culture supernatant	- Inhibition of the biofilm formation without killing the bacteria or inhibiting their growth - Disruption of the *Roseovarius* sp. mature biofilms	This study
